# Injuries and Overuse Injuries in Esports

**DOI:** 10.3390/sports14040127

**Published:** 2026-03-24

**Authors:** Heinz-Lothar Meyer, Ilka Finkemeyer, Christina Polan, Lisa Wienhöfer, Bastian Mester, Marcel Dudda, Manuel Burggraf

**Affiliations:** 1 Department of Trauma, Hand and Reconstructive Surgery, University Hospital Essen, Hufelandstraße 55, 45147 Essen, Germany; ilka.finkemeyer@gmail.com (I.F.); christina.polan@uk-essen.de (C.P.); lisa.wienhoefer@uk-essen.de (L.W.); bastian.mester@uk-essen.de (B.M.); marcel.dudda@uk-essen.de (M.D.); 2 Department of Orthopaedics and Trauma Surgery, BG Klinikum Duisburg, 47249 Duisburg, Germany; 3 Department of Orthopaedics and Trauma Surgery, GFO Kliniken Mettmann-Süd, 40724 Hilden, Germany; info@gfo-kliniken-mettmann-sued.de

**Keywords:** esports, eSport, sport injuries, gaming, esport injuries, e-athletes

## Abstract

Electronic sport (esport) refers to competition in video games. Injuries in esports have hardly been studied so far. A total of 1229 e-athletes of all levels and genres answered a retrospective questionnaire about injuries and overuse damages that occurred in the course of their careers. The average age of the 1229 participants was 23.8 ± 5.5 years. A total of 198 (16.1%) of the e-athletes take part in competitions. The most common injury location was the trunk/spine (319, 26.0%) followed by the wrist region (225, 18.3%). Degenerative and overuse injuries were in the foreground. Professional athletes were injured more frequently than amateur athletes (*p* = 0.006). Tactical shooter players have significantly more injuries than sports game players (*p* = 0.021) and MMO (Massively Multiplayer Online) players (*p* = 0.042). E-athletes are just as susceptible to injury as athletes in traditional disciplines. The high injury rate is certainly not due to acute injuries but to overloading and overuse injuries, with a focus on the thoracocervical area and the upper extremities. Terms such as “Nintenditis”, “gamer’s thumb” and “PlayStation thumb”, which describe injuries caused by repetitive strain, are becoming increasingly common. Injuries in esports should be taken seriously, as they can cause long-term health problems in the event of overuse injuries. Prevention is a critical and promising approach for such a young patient clientele, especially in a sport that is growing so rapidly and is unknown to the majority.

## 1. Introduction

Electronic sport (esport) refers to competition in video or computer games at a professional level. Esport events and tournaments attract a large number of visitors, who can watch the competitions both on site and online [1]. Athletes and teams compete against each other in games of various genres. The 21st century entailed a rapid development of esports. Games such as “League of Legends” and “Dota 2” attracted record numbers of spectators and offered multi-million-dollar prize money that has already surpassed major events in traditional sports [2]. As technology and infrastructure continue to evolve, esports will undoubtedly continue to grow and change, with new games, talents and trends emerging. Esports have gained significant popularity in recent years and are considered one of the fastest growing industries in sports and entertainment [2].

The question of whether esport is a real sport is the subject of current discussions and debates [3]. Esports often require years of training, teamwork and skill development to play at the top level. The inclusion of esports in the Olympic Games is a topic that has received a lot of attention in recent years. Hence, the International Olympic Committee (IOC) has recognized virtual representations of traditional sports as a “sport”, which is seen as a step towards the recognition and regulation of esports by traditional sports institutions. The recognition of esports as a sport by the German Olympic Sports Confederation (DOSB) has not yet taken place [3]. The potential of esports as a global phenomenon is undeniable.

Injuries in esports have hardly been studied to date [4,5]. Progress in sports injury prevention can only be made if research efforts are focused on understanding the implementation context for injury prevention and further developing the evidence base for its efficacy and effectiveness. For this reason, various frameworks have been developed in sports injury research in the past, such as the “Sequence of Prevention” by Mechelen et al. or the “Translating Research into Injury Prevention Practice Framework [TRIPP]” by Finch [6,7]. In all models, a detailed understanding of the etiology, mechanisms, severity and incidence of injuries in the respective sport is a necessary condition for establishing and formally evaluating the effectiveness of injury prevention measures.

Therefore, when analyzing the data that we have collected from e-athletes, we expect to be able to improve the treatment of injuries in such a new and rapidly growing sport and make a decisive contribution to health and injury prevention. With this study, we want to investigate injuries and overuse injuries among e-athletes and highlight possible differences in terms of game level, gender, or genre.

## 2. Materials and Methods

This retrospective cross-sectional epidemiological study was carried out using a questionnaire analogous to previous studies in accordance with the Helsinki Declaration and after review by the responsible ethics committee of the University of Duisburg-Essen (22-10751-BO) [8,9]. Demographic data (gender, date of birth, height, weight, dominant hand, previous illnesses, etc.) and information about injuries and overuse injuries in ten different body regions (head, trunk/spine, shoulder, elbow, wrist, finger, pelvis, thigh, knee, lower leg/foot) were collected. The questionnaire could be answered in German or English.

**Figure 1:**
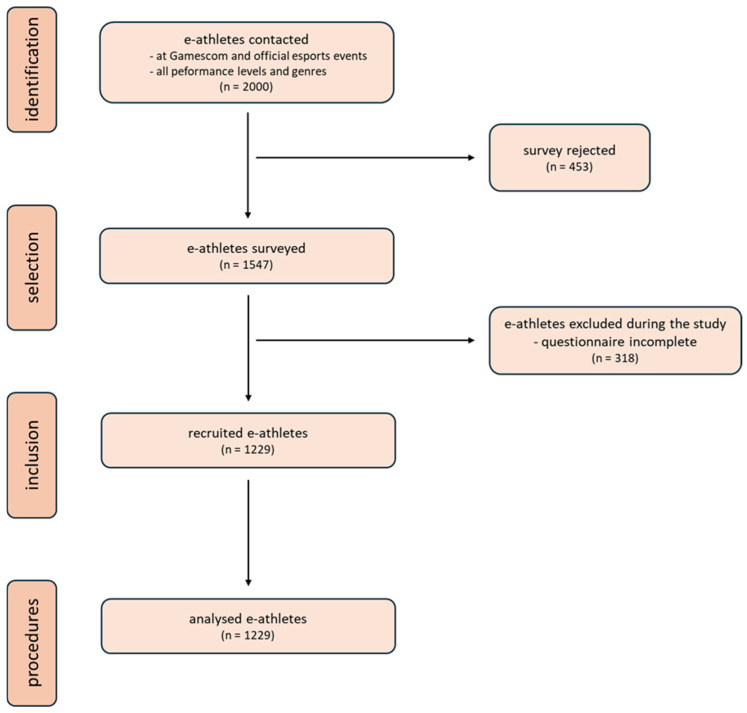
Recruitment flowchart according to the STROBE criteria.

The questionnaire was completed by 1547 active esport athletes of different performance levels and genres. The questionnaires were filled in straightaway online and on paper under the supervision of medical staff who were trained members to answer questions about understanding or to explain medical terms to prevent bias. The questionnaires were filled out voluntarily and anonymously. Participants were randomly selected at official esport events and on The Gamescom in Germany. All completed questionnaires were included in the study. The Gamescom is the world’s largest exhibition for computer and video games and consumer electronics, which takes place annually in Cologne. The recruitment process is shown in Figure 1 according to the STROBE criteria.

**Figure 2:**
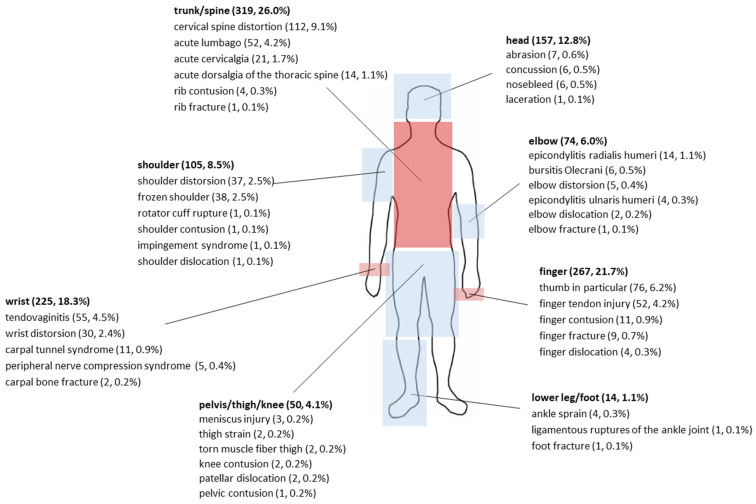
Illustration of injuries in esports by body region.

We defined an injury as tissue damage or other impairment of normal physical function resulting from the practice of sport caused by a rapid or repetitive transfer of kinetic energy, as defined in the International Olympic Committee consensus statement of 2020 [10]. Traditionally, health problems have been divided into those that occur suddenly and those that occur gradually. Sudden-onset health problems were considered to be those caused by a specific identifiable event (e.g., a collision between an athlete and an object causing a fracture) and categorized as injuries. In contrast, gradual-onset problems were considered to be those that lacked a definable sudden triggering event (e.g., tendinopathy caused by repetitive motion). The term “overuse injury” was used in this study to refer to gradually occurring injuries [10]. The classification of the body regions and the distribution of the injuries or overuse injuries to the individual body regions was based on the recommended Sport Medicine Diagnostic Coding System (SMDCS) and the Orchard Sports Injury Classification System (OSICS) [10]. We define professional esport players as those who can earn a living from esports. However, it should be noted that the e-athletes we surveyed did not have a uniform level of prior medical knowledge. This resulted in us clearly defining body areas and injury types, as shown in Figure 2 and Table 3. Demographic data and information on injuries in various parts of the body that occurred during or as a result of esports were collected. In addition, for better statistical comparability with other sports, exposure time was extrapolated with total career duration and weekly training hours, and injuries per 1000 gaming hours were calculated.

### Statistical Analysis

Statistical analysis was performed using the IBM SPSS Statistics 29 software (IBM, Armonk, NY, USA). Descriptive data was analyzed for mean, standard deviation (M ± SD), median and interquartile range (M (IQR)). All values were tested for normal distribution using the Kolmogorov–Smirnov test. Depending on whether a normal distribution was present, the *t*-test or the Mann–Whitney U-test was performed. After the formation of groups (male, female, professionals, amateurs, genres, etc.), the mean values of the individual groups were compared pairwise in post hoc tests according to Bonferroni after an analysis of variance (ANOVA) showed a significant difference between groups to determine which mean values differed significantly from each other. This allowed the specific differences between the groups that led to the significant result to be identified. We considered values of *p* < 0.05 to be significant and *p* < 0.001 to be highly significant. In the study participants, pre-existing conditions were categorized according to the ASA classification. ASA class I corresponds to no pre-existing conditions in the present study. ASA class II corresponds to pre-existing conditions with no or little restriction of daily life, and ASA classification III corresponds to pre-existing conditions with severe restriction of daily life [11].

## 3. Results

In the period from 24 August 2022 to 27 August 2023, 1547 e-athletes were surveyed, 1229 of whom completed the questionnaire in full.

Of the 1229 study participants, 299 (24.3%) were female, 913 (74.3%) were male and 17 (1.4%) were diverse. The average age of the participants was 23.8 ± 5.5 years. The average weight of the 1229 study participants was 80.7 kg ± 19.9. A total of 349 (28.4%) of the study participants are members of a “clan” (organized teams and clubs), while 198 (16.1%) of the e-athletes surveyed take part in competitions. Of these, 110 (9.0%) take part in competitions with prize money. Nineteen (1.5%) of the e-athletes surveyed can earn a living from this. We found that 140 (11.4%) of e-athletes warm up before gaming, with an average warm-up time of 2.3 ± 8.9 min. Further demographic data can be found in Table 1.

On average, the athletes surveyed had 7.4 ± 6.6 years of esports experience, with an average of 21.0 ± 16.4 gaming hours per week. In the available sample, 19.6 injuries per 1000 gaming hours were reported in the overall collective. The exact distribution of injuries per 1000 gaming hours per target group can be seen in Table 2.

### 3.1. Injury Location and Types

The most common injury localization was the trunk/spine (319, 26.0%). Among the injuries to the trunk/spine, cervical spine sprains (112, 9.1%) were reported most frequently, followed by acute lumbar sprain (52, 4.2%).

The second most common injury localization was the wrist (225, 18.3%). The e-athletes suffered most frequently from tendovaginitis (55, 4.5%), followed by wrist contusions (30, 2.4%) and carpal tunnel syndrome (11, 0.9%).

These injury localizations are followed by injuries to the fingers (267, 21.7%) caused by gaming. Finger tendon injuries (52, 4.2%) were mentioned most frequently, followed by finger contusions (11, 0.9%), finger fractures (9, 0.7%) and finger dislocations (4, 0.3%). The thumb (76, 6.2%) was named as the most common region of finger injury caused by gaming. The exact distribution of the reported injuries by injury location can be seen in Figure 2. It was highlighted whether injuries were reported in the corresponding region, and if these were specified, this was also reported. The more distant the injury region is from the upper extremity and trunk/spine, the fewer injuries are reported in the body region. Injuries to the upper extremities, in particular the wrists, were reported as the most common cause of gaming time loss. Table 3 shows the exact length of the gaming time loss based on the injured body region.

### 3.2. Comparison of the Groups

#### 3.2.1. Competitors vs. Non-Competitors

In the comparison of the groups, significantly more e-athletes who participate in competitions (71; 35.9%) than e-athletes who do not participate in competitions (248; 24.1%) had to pause gaming due to trunk/spine injuries after gaming (*p* < 0.001, OR 1.8). Likewise, significantly more competitors (22; 11.1%) than non-competitors (68; 6.6%) had to pause gaming due to cervical spine sprain after gaming (*p* = 0.036, OR 1.8).

When comparing these groups, significantly more competitors (41; 20.7%) than non-competitors (74; 7.2%) had to pause gaming due to a hand injury (*p* < 0.001, OR 3.4). Significantly more competitors (50; 25.3%) than non-competitors (125; 12.1%) also had to pause gaming due to a finger injury (*p* < 0.001, OR 2.5). Significantly more competitors (28; 14.1%) than non-competitors (64; 6.2%) had to pause gaming due to finger strain after gaming (*p* < 0.001, OR 2.5), pause gaming due to pain in the thumb after gaming (*p* = 0.023, OR 1.1), pause gaming due to a finger contusion (*p* = 0.004, OR 6.4), pause gaming due to a finger tendon injury (*p* < 0.001, OR 4.1), or pause gaming due to a finger dislocation (*p* = 0.015, OR 15.8). Significantly more competitors (73; 36.9%) than non-competitors (152; 14.7%) had to pause gaming due to a wrist injury (*p* < 0.001, OR 3.4). Significantly more competitors (18; 9.1%) than non-competitors (37; 3.6%) had to pause gaming due to wrist tendinitis (*p* = 0.002, OR 2.7), forearm nerve compression syndrome after gaming (*p* = 0.032, OR 7.9), or wrist contusion after gaming (*p* < 0.001, OR 4.2). There were no significant differences between the two groups in the area of the lower extremities (*p* > 0.05, OR 1.0).

#### 3.2.2. Professionals (Earning Their Living Through Gaming) vs. Amateurs (Non-Professionals)

Significantly more professional athletes (6; 31.6%) than amateur athletes (109; 9.0%) had to pause esports due to injury/overuse in general (*p* = 0.006, OR 4.7). Significantly more professional athletes (9; 47.4%) than amateur athletes (166; 13.7%) had to pause gaming due to an injury (*p* < 0.001, OR 5.7) or overuse (professional athletes 9; 47.4%/amateur athletes 83; 6.9%; *p* < 0.001, OR 12.2) of their fingers after gaming. The thumb was significantly more frequently affected by this in professional athletes (4; 21.1%) than in amateur athletes (72; 6.0%) (*p* < 0.001, OR 4.2). The type of injury due to which professional athletes had to pause gaming was significantly more often finger dislocation (*p* = 0.001, OR 2.9), wrist pain (*p* < 0.001, OR 6.4) or tendinitis (*p* < 0.001, OR 10.9).

Furthermore, significantly more professional athletes (4; 21.1%) than amateur athletes (70; 5.8%) had to pause gaming due to an elbow injury after gaming (*p* = 0.024, OR 4.3). Professional athletes were significantly more frequently affected by tennis elbow (*p* = 0.018, OR 11.7).

Significantly more professional athletes (5; 26.3%) had to take a break from gaming due to a shoulder injury after gaming than amateur athletes (100; 8.3%) (*p* = 0.018, OR 4.0). Significantly more professional athletes (3; 15.8%) than amateur athletes (15; 1.2%) were affected by a shoulder sprain (*p* = 0.002, OR 14.9).

There were no significant differences in the other body regions.

### 3.3. Comparison of the Genres

**Figure 3:**
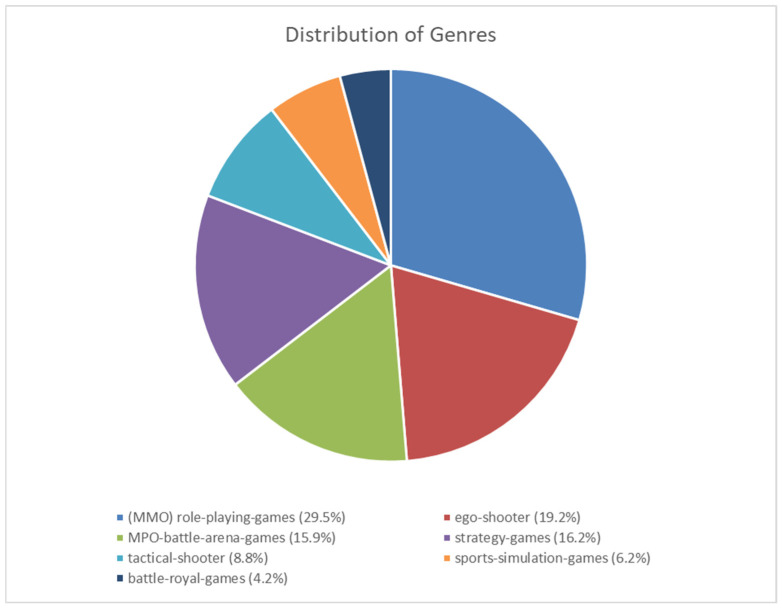
Distribution of the interviewed esport players across the individual genres.

Figure 3 shows the distribution of the esport players surveyed across the individual genres.

For better comparability, only the esport players (n = 308) who only play one genre are shown in the graph. When comparing the esport players of the individual genres, significant differences were found between the gaming time loss due to injuries/overuse in general (*p* = 0.02).

Significant differences were also found in gaming time loss due to injuries (*p* = 0.016) and overuse of the finger (*p* = 0.041), wrist contusions (*p* = 0.008) and injuries and pain to the trunk/spine (*p* = 0.012) between the individual genres.

Tactical shooter players (14; 51.9%) had significantly more injuries or pain in the back, neck and thorax than sports game players (1; 5.3%) (*p* = 0.021, OR 0.1) and MMO (Massively Multiplayer Online) players (18; 19.8%) (*p* = 0.042, OR 0.2).

### 3.4. Comparison of Gender-Specific Differences

Of the 1229 study participants, 299 (24.3%) were female, 913 (74.3%) were male, and 17 (1.4%) were diverse. Significantly more male e-athletes (20, 2.2%) had to take a break from esports due to tendon injuries in the hand than female e-athletes (0, 0%) (*p* = 0.021, OR 2.8). Significantly more male e-athletes (20, 2.2%) suffered shoulder injuries from eports than female e-athletes (15, 5.0%) (*p* = 0.048, OR 2.4).

Male (64, 7.1%) (*p* = 0.036) and female (20, 6.7%) (*p* = 0.012) e-athletes reported significantly more spinal disk problems after gaming than the group of diverse e-athletes (3, 17.6%). There are no significant differences between male and female e-athletes in this category (*p* = 1.000).

There are no significant gender-specific differences in any other categories, as illustrated in Table 4.

## 4. Discussion

Esports are becoming extremely popular worldwide. Nevertheless, there are still few studies on injuries and overuse in esports [12,13].

### 4.1. Epidemiology

It appears that it is predominantly men (913; 74.3%) who practice esports. The average age for esports is relatively young at 23.8 ± 5.5 years. This data is consistent with recent press releases. Women are still strongly under-represented in esports. This is especially true for professional e-athletes, although women make up about 30% of the audience [14]. Due to the growing popularity of esports, there are more and more competitions. In our study, 16.1% (198) of the e-athletes surveyed take part in competitions, and 1.5% (19) of those can earn a living from it.

### 4.2. Health and Esports

The majority of participants in our study reported no pre-existing medical conditions (990; 80.6%), and more than half engaged in a recreational sport in addition to esport (696; 56.6%). In contrast, a study conducted by DiFrancisco-Donoghue et al. showed that e-athletes are significantly less active and have a higher body fat percentage, lower lean body mass and lower bone mineral content than non-athletes [15]. Lam et al. were able to show that the duration of an esports career was not associated with BMI or body fat percentage [4]. In general, it can be said that studies on esports games have mainly focused on the violent tendencies and aggressive behavior of users, while less attention has been paid to the positive effects on the athletic health of young people [16].

### 4.3. Social Integration of Esports

The literature also assumes that esports have a socially isolating effect on e-athletes, especially during and after the COVID-19 pandemic [17]. In our study, we were able to show that more and more e-athletes are members of a club (119; 9.7%) or a clan (349; 28.4%). Esport has great potential to be an inclusive sport. Some athletes with physical disabilities can compete well in esports with non-disabled e-athletes without a physical disadvantage. However, as portrayed in the media, esport does not yet appear to be a truly inclusive sport. Other positive effects of esports can be an improvement in cognitive abilities to increase athletic performance and an improvement in visual memory and reaction time [18]. Regularly playing action video games is known to improve visual–spatial awareness and related skills [19].

### 4.4. Injuries in Esports

In the available sample, 19.6 injuries and overuse injuries were reported per 1000 gaming hours in the overall collective. The athletes surveyed had an average of 7.4 ± 6.6 years of esports experience, with an average of 21.0 ± 16.4 gaming hours per week. This data shows a surprisingly high rate of injuries per 1000 h of gaming. Other sports such as soccer show an injury rate of 2–7 injuries per 1000 h of play in training and around 35 injuries per 1000 h of play in competition [20]. In rugby, injury rates of 101.5 to 119.8 per 1000 h of play are reported, in basketball, 12.59 injuries are reported, and in archery, a rate of 0.005 injuries per 1000 h of play is reported [21,22,23]. The comparatively high rate of injuries and overuse injuries in esports indicates a high level of physical demand and many repetitive movement patterns in gaming, which predominantly lead to overuse injuries. The injury rate is comparable to that of contact sports, although the focus in contact sports is on acute injuries rather than overuse injuries.

### 4.5. Injury Locations and Types in Esports

The most common injury localization in the present study was the trunk/spine (319, 26.0%), with cervical spine sprains (112, 9.1%) being the most common. This was followed by the wrist (225, 18.3%), hand and fingers. The e-athletes suffered most frequently from tendovaginitis (55, 4.5%) and carpal tunnel syndrome (11, 0.9%). This may be because e-athletes spend between 12 and 15 h a day practicing dynamic and repetitive movements with a mouse and keyboard to play at a top level, usually in conjunction with a suboptimal posture. This also results in the accumulation of overuse injuries in the cervicothoracic region observed in our study. Sitting for hours in an unfavorable position often leads to these postural problems, tension and the above-mentioned overuse injuries. E-athletes perform both isotonic and isometric contractions of the upper limbs to perform up to 400 fine motor movements per minute or to stabilize the wrist–elbow–shoulder girdle. These long fine motor movements of the upper limbs are predisposed to the development of the above-mentioned injuries due to repetitive strain. Further consequences can be chronic tendinopathies, myofascial pain and compression neuropathies [15,24].

The terms “Nintenditis”, “gamer’s thumb” and “PlayStation thumb” have been coined in case reports to describe repetitive strain injuries caused by prolonged video game play [25]. A study of 120 Malaysian university students suggests that the symptoms of carpal tunnel syndrome are not only promoted by repetitive strain injury, but can also be exacerbated by playing, as was also shown in our study [26]. A study by Prado et al. found that constant computer use in esports leads to repetitive strain on the superficial and deep flexor muscles of the fingers, which squeeze and irritate the median nerve in the carpal tunnel, causing carpal tunnel syndrome (CTS) [27]. Peripheral neuropathies were also found in a study by Yoon et al. [28].

Lee et al. [29] found a 73–88% prevalence of musculoskeletal disorders in musicians. The recognition of pathologies in musicians has led to the development of specialized multidisciplinary teams that include physiotherapists, hand surgeons and psychologists [29]. Due to the strong growth of esports and its increasing popularity, the number of acute injuries, in particular repetitive strain injuries, tendinopathies and compression neuropathies, in esports will rise sharply. The spectrum of patients ranges from amateur athletes to elite professionals. As shown in our study, significantly more e-athletes who participate in competitions (71; 35.9%) than e-athletes who do not participate in competitions (248; 24.1%) suffer injuries, particularly to the trunk/spine (*p* < 0.001), cervical spine (*p* = 0.036), hand (*p* < 0.001) and fingers (*p* < 0.001). Professional athletes (6; 31.6%) were also significantly more likely to have to take a break from esports due to injury/overuse than amateur athletes (109; 9.0%) (*p* = 0.006). The fingers (*p* < 0.001) and thumb (*p* < 0.001) were particularly affected. This is probably related to the excessive training and performance pressure of competitors and professional athletes. In particular, the upper extremities and the trunk/spine and neck muscles are strained. In the present study, we were able to show that tactical shooter players (14; 51.9%) have significantly more injuries or pain in the back, neck and thorax than sport simulation players (1; 5.3%) (*p* = 0.021) and MMO players (18; 19.8%) (*p* = 0.042). This is probably due to the type of game and the physical demands of the individual genres. Tactical shooter games certainly have faster and more repetitive movement patterns than sport simulations, for example. At present, there are only a few organizations that deal with the treatment and rehabilitation of esport injuries. Healthcare facilities should be prepared to meet this need. The establishment of multidisciplinary treatment teams, analogous to the multidisciplinary teams mentioned above with hand surgeons, orthopedic surgeons, physiotherapists, occupational therapists and psychologists, may be useful for this unique patient group to ensure appropriate care and patient satisfaction, especially for elite athletes.

### 4.6. Gender-Specific Differences in Esports

In many sports, there is a gender-specific difference, especially when it comes to injuries and overuse injuries [30]. Female athletes are at higher risk for certain types of injuries, like anterior cruciate ligament (ACL) injuries, bone stress injuries, concussions, and non-traumatic shoulder instability [31]. But this really depends on the type of sport and the physical conditions and demands of the sport [32].

In the present study, gender-specific differences in injuries and overuse injuries were only found in a few categories. This suggests that there are no real gender-specific differences in esports and also suggests the inclusive nature of esports.

### 4.7. Limitations of This Study

Acute injuries that occurred due to overuse injuries, such as fatigue fractures, were counted as acute injuries in this study. The study participants were unable to differentiate whether a health problem resulted from a clear acute mechanism or a clear repetitive/overuse mechanism, or whether it was a mixture of both elements. This was because of a lack of diagnostics and medical knowledge to categorize this accurately, as required by the IOC consensus decision [10]. This could be better differentiated and categorized through expanded diagnostics and more accurate questioning in follow-up studies.

The present study investigates fewer health problems. The focus of the present study is on injuries and overuse injuries and examines fewer general health problems as a consequence of esports, as defined in the IOC consensus paper. This could be better investigated and included in future studies to find possible associations or differences between injuries and health problems.

A strength of this study is the high number of participants, which strengthens the present results.

## 5. Conclusions

Esport is a sporting competition with video games and is recognized by established associations of organized sports in over 60 nations (e.g., South Korea, USA, Brazil, China, France, etc.) and is partially supported by the state [33,34]. This will require the development of many regulatory mechanisms in the future, such as fair play or regulations on sports doping [35].

It may come as a surprise, but e-athletes are just as susceptible to injury as athletes in traditional disciplines [20]. The high injury rate is certainly not due to acute injuries but to overuse injuries with a focus on the thoracocervical area and the upper extremities, especially in the wrist, hand and finger area. Acute injuries also occur, with a focus on the upper extremities. Terms such as “Nintenditis”, “gamer’s thumb” and “PlayStation thumb”, which describe injuries caused by repetitive strain, are becoming increasingly common [25]. In addition to the negative consequences of esports, various research studies have already identified many positive effects. The willingness of young people to participate in sport and fitness can increase, offering great potential for an inclusive sport. Many physiological characteristics can be improved, such as the improvement of cognitive abilities, visual memory, reaction time and spatial awareness [16,18,19].

It is important to emphasize that injuries in esports should be taken seriously, as they can affect athletes’ performance and cause long-term health problems in the event of overuse injuries. Prevention is an essential and promising approach for such a young patient clientele. An ergonomic gaming environment (e.g., gaming chair, table height, distance from the screen, etc.) and gaming equipment (ergonomic mouse and keyboard, etc.) can be preventive measures to significantly reduce acute but above all overuse injuries. Future studies must further investigate and confirm the effectiveness of these preventive measures and explore possible correlations with injury rates. Raising awareness of this issue and multidisciplinary cooperation in the prevention, treatment and rehabilitation of e-athletes between orthopedic surgeons, hand surgeons, physiotherapists, occupational therapists and psychologists are important, especially in a sport that is growing rapidly and is unknown to the majority. Esports will certainly continue to grow and increase in popularity.

## Figures and Tables

**Table 1:** Demographic data of the e-athletes.

		E-Athletes	
		Number (n)	Relative Frequency (%)
gender	male	913	74.3%
	female	299	24.3%
	diverse	17	1.4%
dominant hand	right	1092	88.9%
	left	137	11.1%
members of esports club	yes	119	9.7%
	no	1110	92.3%
members of a clan	yes	349	28.4%
	no	880	71.6%
take part in esports competitions	yes	198	16.1%
	no	1031	83.9%
warm-up before gaming	yes	140	11.4%
	no	1089	88.6%
already been injured by a gaming object	yes	131	10.7%
	no	1098	89.3%
plays a recreational sport	yes	696	56.6%
	no	533	43.4%
pre-existing conditions	ASA class 1	990	80.6%
	ASA class 2	142	11.6%
	ASA class 3	28	2.3%
	no ASA class given	69	5.5%

**Table 2:** Injuries per 1000 h of esports.

Group	n	Injuries/1000 Esport Hours
total collective	1229	19.6
competitors	198	27.7
non-competitors	1031	17.1
male	913	18.4
female	299	23.4
diverse	17	28.6
earns living with esports	19	34.5
no income from esports	1210	17.1
MPO-battle-arena players	49	26.0
battle-royal players	13	35.6
ego-shooter players	59	16.8
tactical shooter players	27	16.0
sport simulations players	19	44.8
strategy gamers	50	17.3
(MMO) role-playing gamers	91	21.2

**Table 3:** Number of weeks that led to gaming time loss due to injuries.

Injury Location	Time Loss (Weeks ± SD)
finger	0.08 ± 0.60
wrist	0.21 ± 0.92
elbow	0.04 ± 0.32
shoulder	0.04 ± 0.47
trunk/spine	0.05 ± 0.38
head	0.02 ± 0.20
pelvis/thigh/knee	0.00 ± 0.05
lower leg/ankle/foot	0.01 ± 0.22

**Table 4:** Gender-specific differences (male vs. female).

Category	Male (n (%))	Female (n (%))	Group Comparison (*p*-Value)
break in esports due to injury/overuse injury	86 (9.4%)	26 (8.7%)	0.388
wrist injuries	169 (18.5%)	53 (17.7%)	0.950
elbow injuries	55 (6.0%)	18 (6.0%)	1.000
neck strain	66 (7.2%)	22 (7.4%)	0.614
head injuries	113 (12.4%)	41 (13.7%)	0.570
pelvis/thigh/knee injuries	39 (4.3%)	10 (3.3%)	0.525
lower leg/ankle/foot injuries	13 (1.4%)	1 (0.3%)	0.350

## Data Availability

Dataset is available on request from the authors. The data are not publicly available due to privacy.
